# Trends in Research on Plant Genetic Resources: Insects, Plant Diseases, and Genetic Diversity

**DOI:** 10.3390/plants15101471

**Published:** 2026-05-12

**Authors:** Seong-Hoon Kim, Inchan Choi

**Affiliations:** 1 National Agrobiodiversity Center (Genebank), National Institute of Agricultural Sciences, Rural Development Administration, Jeonju 54875, Republic of Korea; 2 Division of Agricultural Engineering, National Institute of Agricultural Sciences, Rural Development Administration, Jeonju 54875, Republic of Korea

Plant genetic resources constitute the biological foundation of crop improvement and are essential for sustaining global food security. As agricultural production faces escalating pressures from climate instability, evolving pathogens and pests, habitat degradation, and narrowing breeding pools, the strategic conservation and effective utilization of genetic diversity have become more urgent than ever [1]. The genetic diversity preserved within wild relatives, landraces, traditional cultivars, and gene bank collections provides an invaluable reservoir of adaptive traits that can be exploited to improve biotic resistance, productivity, and resilience in crop species [2]. In this context, this Special Issue of *Plants*, titled “Trends in Research on Plant Genetic Resources: Insects, Plant Diseases, and Genetic Diversity”, brings together four research articles and one review articles that collectively illustrates how contemporary plant genetic resources research is moving beyond simple conservation towards a more integrated paradigm that combines molecular characterization, population analysis, gene bank rationalization, and breeding-oriented deployment of diversity. The main objective of this Special Issue is to associate conservation and utilization of valuable genetic resources with their underlying genetic basis, thereby enabling innovative strategies for biotic stress mitigation and promoting crop resilience.

Recent advances in high-throughput genotyping, phenomics, bioinformatics, and spatial analysis have substantially expanded the capacity to explore and utilize plant genetic resources more effectively [3,4]. Modern breeding increasingly relies on integrated approaches that combine molecular markers, genome-wide diversity assessment, georeferenced conservation data, and phenotypic evaluation to identify valuable genetic variation and improve selection efficiency. Consequently, gene banks are evolving from passive repositories of germplasm into dynamic research infrastructures that support genetic discovery, trait introgression, long-term management of germplasm diversity, and global germplasm exchange [5,6]. These advancements are significant for broadening the genetic bases of crops and accelerating the development of resilient cultivars adapted to future agricultural challenges.

The contributions included in this Special Issue demonstrate that effective utilization of plant genetic resources is fundamentally dependent upon the availability of robust and informative characterization tools. One study reported the development of SSR markers for the tropical Andean fruits *Solanum quitoense* and *Solanum betaceum* and revealed a relatively narrow genetic base and substantial cultivar homogeneity, particularly in *S. betaceum* [7]. These findings highlight a persistent change challenge in underutilized crops, where breeding process is frequently constrained by limited genetic genomic resources and restricted diversity within germplasms. By clarifying the relationships among cultivated materials, landraces, and wild relatives, the study establishes an important foundation for future molecular breeding and the broadening of the genetic base in these species.

A complementary breeding-oriented study investigated trait segregation, heterosis, and inheritance patterns in F1 progeny derived from Chinese white pear and Western pear parents [8]. The results revealed extensive phenotypic variation in major fruit quality and growth traits, while identifying juvenile characteristics with potential utility for early-stage selection. This work illustrates how the phenotypic diversity generated through hybridization can be systematically exploited to guide parental selection and accelerate breeding progress.

Similarly, this issue has contributions focusing on strengthening germplasm characterization in European plum through the development of SSR genotyping capable of discerning 242 unique genotypes [9]. In perennial fruit crops such as European plum, extensive synonymy, homonymy, and clonal propagation often complicate cultivar identification; therefore, the availability of an efficient and standardized molecular marker system is particularly important. The establishment of a standardized allele atlas represents an important advance for perennial fruit crop research and management, as accurate cultivar identification is essential for minimizing redundancy, preventing misidentification, and improving traceability within breeding programs and gene bank collections.

The conservation dimension of plant genetic resources is addressed through a study [10] integrating geobotanical surveys, remote sensing, DNA barcoding, and SSR marker analysis to assess rare and endangered apricot populations of Kazakhastan. The findings represent considerable genetic variability, but also pronounced population isolation, geographic structuring, and localized vulnerability to environmental threats. The ecological context, with molecular evidence, demonstrates how conservation priorities can be more effectively directed toward genetically distinct, isolated, and threatened populations.

Collectively, the studies highlight several emerging trends in current plant genetic resources research. Most notably, they reflect an increasingly integrative research framework in which genomics, molecular markers, phenotyping, geobotany, and spatial analyses are applied in a complementary manner to characterize and utilize diversity more effectively. This trend represents a broader transition from passive germplasm conservation toward data-driven and application-oriented characterization, where genetic and phenotypic information is generated not only to document diversity, but to also support germplasm curation, conservation prioritization, and breeding. Further, these contributions emphasize that both major crops and underutilized species require equally rigorous molecular and analytical resources, particularly taxonomical complexity, or fragmented populations constrain genetic improvement.

Overall, this Special Issue emphasizes that the future value of plant genetic resources lies not merely in their preservation, but in their transformation into analytically resolved, biologically interpretable, and breeding-relevant resources. In the context of climate change, emerging pests and diseases, and increasing food demand. Therefore, stronger integration among gene banks, breeding programs, and molecular genetic research will be essential to translate conserved diversity into practical crop improvement and global food security.
